# Supplementary material for: Prospective Analysis of B Lymphocyte Subtypes, before and after Initiation of Dialysis, in Patients with End-Stage Renal Disease
Source: Life (Basel). 2023 Mar 23;13(4):860. doi: 10.3390/life13040860 (PMC10146774; doi:10.3390/life13040860)
Supplement: Supplementary file 1 [file life-13-00860-s001.zip › life-2247522-supplementary.pdf]

Supplementary Table S1. Clinical and laboratory findings of ESRD-T0 patients, differences between patients started on CAPD or HD

	**CAPD**	**HD**	**p-values**
N	18	22	
Age(yrs)	55.00(24)	62.00(15)	NS
WBC (K/μL)	6200.00(1950)	6740.00(3543)	NS
Neutrophils (%)	67.2(8.5)	69.8(14.3)	NS
Lymphocytes (%)	22.4(8.6)	21.45(13.1)	NS
NLR	3.2(0.9)	3.8(1.5)	NS
Monocyte (%)	8.30(1.8)	7.1(2.3)	NS
Eosinophils (%)	30(2.6)	2.1(1.9)	NS
Basophils (%)	0.70(0.2)	0.5(0.4)	NS
Neutrophils (K/μL)	3700.00(1050)	6250.00(3725)	NS
Lymphocytes (K/μL)	1500.00(750)	1200.00(1128)	NS
Monocytes (K/μL)	500.00(250)	550.00(180)	NS
Eosinophils (K/μL)	200.00(250)	150.00(100)	NS
Basophils (K/μL)	0.00(21)	0.00(100)	NS
CRP	1.2(5.8)	1.9(7.2)	NS
Serum Total Protein (g/dL)	6.9(1.1)	6.7(1.4)	NS
Serum Albumin (g/dL)	3.9(0.8)	3.7(0.5)	NS
Intact PTH	262(386)	314(585)	NS
Cholesterol (mg/dL)	174.00(79)	149.00(48)	NS
Triglycerides (mg/dL)	172.00(89)	118.00(159)	NS
LDL (mg/dL)	93.00(50)	88.00(35)	NS
HDL(mg/dL)	41.00(10)	40.50(13)	NS
Troponin (mg/dL)	0.1(5.5)	0.12(8.5)	NS

Abbreviations: ESRD-T0: End Stage Renal Disease at T0, WBC: White Blood Cells, CRP: C-Reactive Protein, PTH: Parathyroid hormone, LDL: Low Density Lipoprotein, HDL: High Density Lipoprotein, NS: Non-Significant

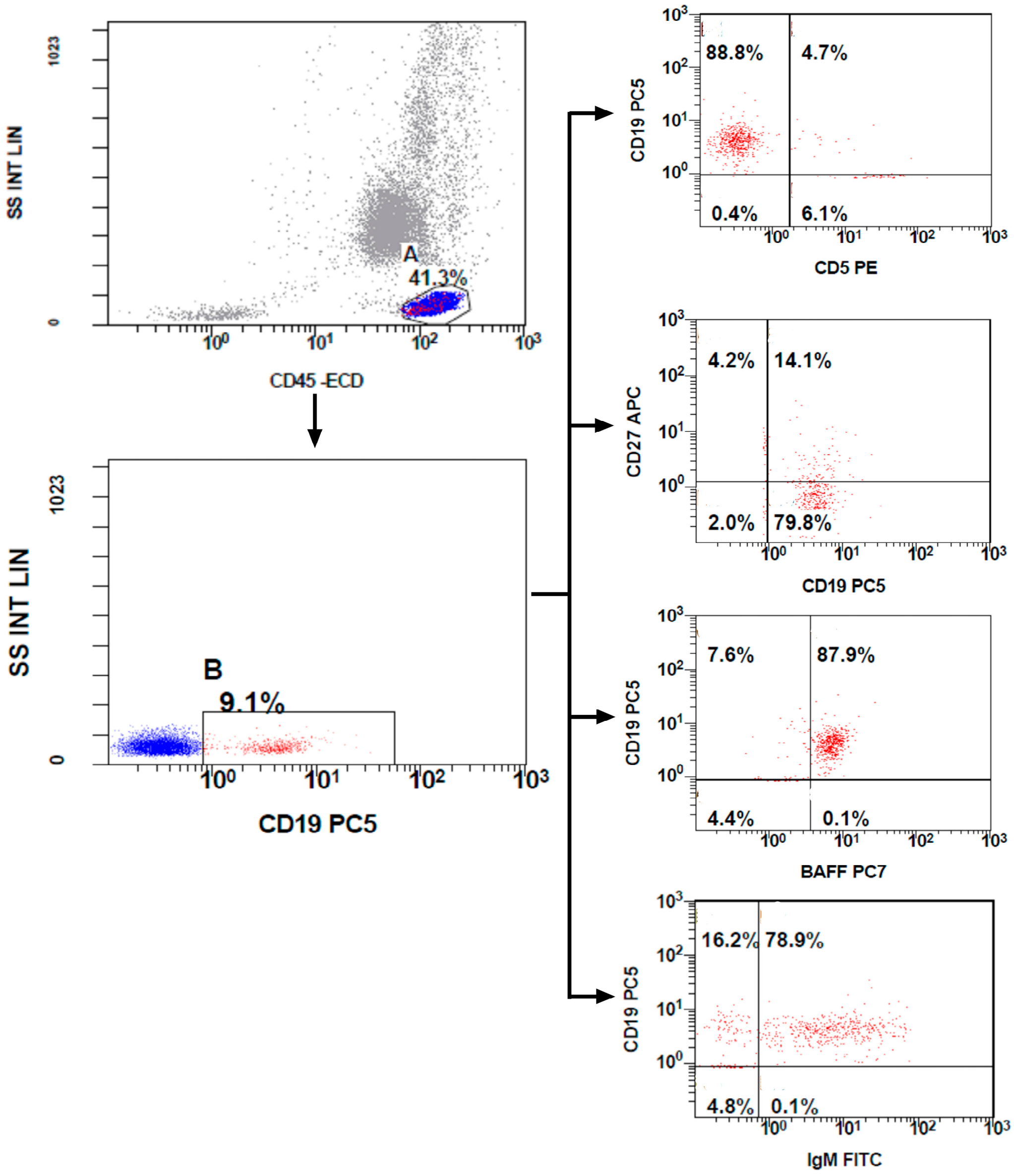

Supplementary Figure S1. Gating strategy for CD45+ cells, CD19+ lymphocytes, and consequently for CD19+CD5+, CD19+CD27+, CD19+BAFF+ and CD19+IgM+ subtypes

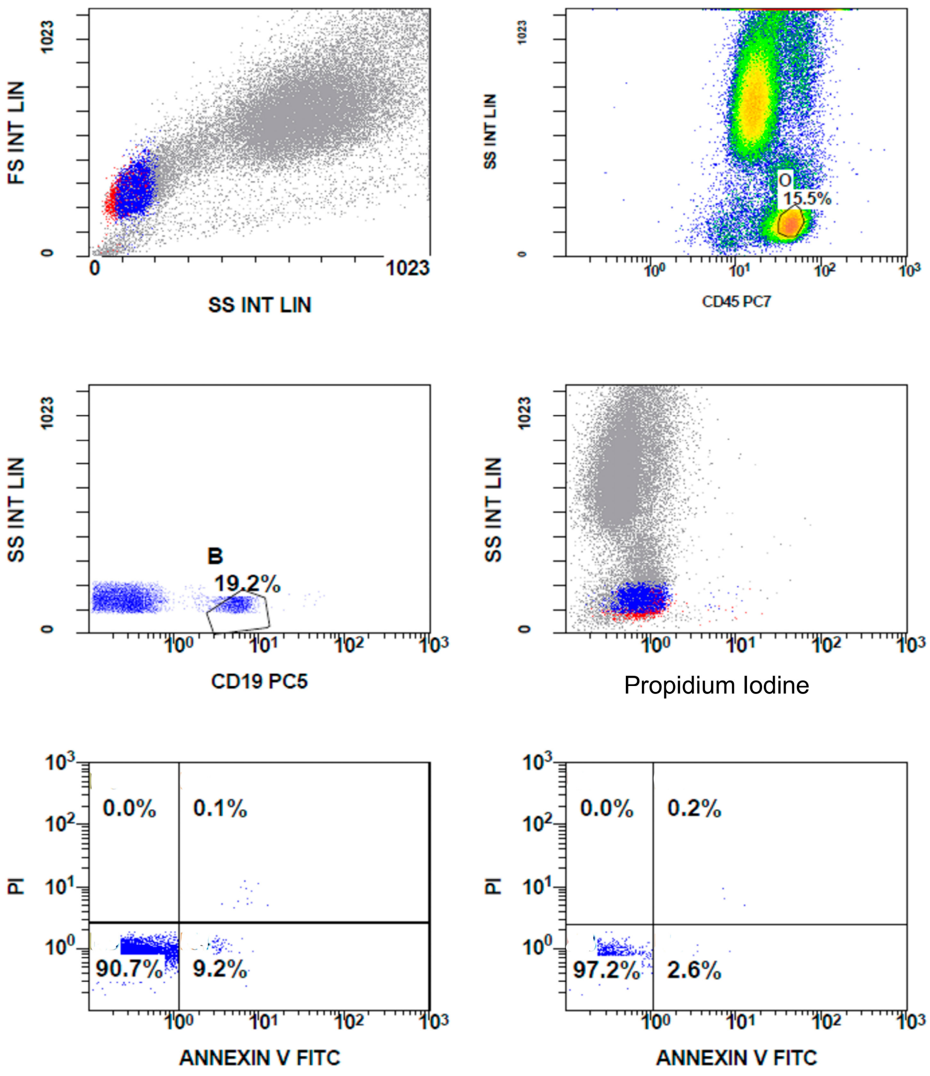

Supplementary Figure S2. Gating strategy for apoptotic total lymphocytes and B lymphocytes.
